# Maternal Immune Activation Sensitizes Male Offspring Rats to Lipopolysaccharide-Induced Microglial Deficits Involving the Dysfunction of CD200–CD200R and CX3CL1–CX3CR1 Systems

**DOI:** 10.3390/cells9071676

**Published:** 2020-07-12

**Authors:** Katarzyna Chamera, Magdalena Szuster-Głuszczak, Ewa Trojan, Agnieszka Basta-Kaim

**Affiliations:** Laboratory of Immunoendocrinology, Department of Experimental Neuroendocrinology, Maj Institute of Pharmacology, Polish Academy of Sciences, 12 Smętna St., 31-343 Kraków, Poland; chamera@if-pan.krakow.pl (K.C.); szuster@if-pan.krakow.pl (M.S.-G.); trojan@if-pan.krakow.pl (E.T.)

**Keywords:** prenatal immune challenge, lipopolysaccharide, CX3CL1–CX3CR1, CD200–CD200R, microglia, two-hit, schizophrenia

## Abstract

Early life challenges resulting from maternal immune activation (MIA) may exert persistent effects on the offspring, including the development of psychiatric disorders, such as schizophrenia. Recent evidence has suggested that the adverse effects of MIA may be mediated by neuron–microglia crosstalk, particularly CX3CL1–CX3CR1 and CD200–CD200R dyads. Therefore, the present study assessed the behavioural parameters resembling schizophrenia-like symptoms in the adult male offspring of Sprague-Dawley rats that were exposed to MIA and to an additional acute lipopolysaccharide (LPS) challenge in adulthood, according to the “two-hit” hypothesis of schizophrenia. Simultaneously, we aimed to clarify the role of the CX3CL1–CX3CR1 and CD200–CD200R axes and microglial reactivity in the brains of adult offspring subjected to MIA and the “second hit” wit LPS. In the present study, MIA generated a range of behavioural changes in the adult male offspring, including increased exploratory activity and anxiety-like behaviours. The most intriguing finding was observed in the prepulse inhibition (PPI) test, where the deficit in the sensorimotor gating was age-dependent and present only in part of the rats. We were able to distinguish the occurrence of two groups: responsive and non-responsive (without the deficit). Concurrently, based on the results of the biochemical studies, MIA disrupted mainly the CD200–CD200R system, while the changes of the CX3CL1–CX3CR1 axis were less evident in the frontal cortex of adult non-responsive offspring. MIA markedly affected the immune regulators of the CD200–CD200R pathway as we observed an increase in cortical IL-6 release in the responsive group and IL-4 in the non-responsive offspring. Importantly, the “second hit” generated disturbances at the behavioural and biochemical levels mostly in the non-responsive adult animals. Those offspring were characterized both by disturbed PPI and “priming” microglia. Altogether, the exposure to MIA altered the immunomodulatory mechanisms, including the CD200–CD200R axis, in the brain and sensitized animals to subsequent immunological challenges, leading to the manifestation of schizophrenia-like alterations.

## 1. Introduction

Early life challenges, resulting from maternal immune activation (MIA), can have long-lasting consequences in the offspring. Those changes include increased susceptibility to adverse postnatal psychological outcomes, such as cognitive impairments and psychiatric disorders [1]. According to epidemiological studies, MIA, e.g., in the form of bacterial or viral infection, malnutrition, or stress, exerts profound effects on foetal development in the uterus and increases the risk of brain pathologies, including schizophrenia [2,3,4,5]. Exposure to MIA is capable of enhancing the pro-inflammatory response in the three maternal-foetal compartments, namely, the placenta, the amniotic fluid and the foetus, particularly in the foetal brain [6,7]. Nevertheless, the pathophysiological mechanisms by which MIA-induced inflammatory events leading to neurodevelopmental and behavioural changes in adult offspring are not completely understood.

Microglia are the immune-competent cells of mesodermal origin with the ability to transform their morphological phenotype and the capacity to migrate, proliferate, and phagocytose [8]. Recent evidence supports a role for microglia as a critical cellular component linked to impairments in brain development and, consequently, aberrant postnatal behaviours [9,10]. The elevation in levels of pro-inflammatory cytokines has been observed in the cerebrospinal fluid or post-mortem brain tissue of patients with schizophrenia [11,12], promoting this phenomenon. A study using positron emission tomography revealed that activated microglia are present within the first five years of schizophrenia onset in individuals with the disease [13]. Additionally, substantially increased numbers of microglia have been detected in the anterior cingulate cortex and mediodorsal thalamus of patients who had committed suicide during acute psychosis [14]. Microglial cells are responsible for the induction of an innate immune response by receiving and propagating inflammatory signals. Under physiological conditions, microglia continuously survey the brain microenvironment and show a highly sensitive response to potential challenges to homeostasis [15]. When activated, microglia secrete various cytokines, engage in phagocytosis, regulate astrocyte pathogenic activity, and/or promote repair by secreting growth factors [9]. Consequently, these cells play a dual role in the brain and are characterized by various immune and cytokine profiles. Although the heterogeneity of microglial activation states is documented [16,17,18], generally, two phenotypes have been highlighted: (1) the classical, pro-inflammatory phenotype, which includes MHCII, CD40, iNOS, IL-1β, TNF-α, and IL-6, and (2) the alternative phenotype that is mainly characterized by the anti-inflammatory mediators ARG1, IGF-1, TGF-β, IL-4, and IL-10 [19]. Of particular relevance to the present study, a primed activation state may be induced in microglia as a consequence of MIA and it may aggravate the neuronal dysfunction and behavioural abnormalities observed in the offspring [20,21]. A primed state has been associated with morphological and phenotypic changes [22], as well as excess cytokine release upon exposure to a pro-inflammatory stimulus, such as lipopolysaccharide (LPS) [20,21,23]. This observation may suggest an alternative “two-hit” explanation of the aetiology of schizophrenia. In this concept, a “first hit”, which occurs during prenatal life (i.e., MIA) and involves priming the microglia of the offspring, increases the vulnerability to another insult. The “second hit”, which might occur later in life, may lead to anomalous or exaggerated microglial activation and, finally, the onset of the disease [24].

Microglia activity is regulated by various mechanisms within the central nervous system (CNS), including the exchange of signals linking microglia with other brain cells, particularly neurons [25]. This communication determines normal neurodevelopmental processes and blood–brain barrier integrity [26,27]. Malfunctions of this interaction upregulate the phagocytic activity, mobility, and release of pro-inflammatory factors by microglia [10]. In these mechanisms, the specialized endogenous protein systems CX3CL1–CX3CR1 and CD200–CD200R play a crucial role and represent unique ligand–receptor axes. 

Since neurons are the main source of CX3CL1 (also known as fractalkine), its expression is remarkably higher in the brain than in the periphery [28], suggesting a unique role for CX3CL1 in the CNS. This protein is the only ligand of CX3CR1, a receptor present on microglial cells. In addition to the main function of CX3CL1, which is the induction of chemotaxis and cell adhesion, the CX3CL1–CX3CR1 axis regulates the activation and proper function of microglial cells [29] and neuronal survival [30]. This system mainly controls the release of inflammatory factors and, consequently, the resolution of inflammation [31]. 

The CD200 glycoprotein is a surface antigen with a critical role in regulating and maintaining the resting state of microglial cells [32]. It is expressed on neurons, while the CD200 receptor (CD200R) is present almost exclusively on myeloid cells. Disturbances in the CD200–CD200R communication stimulate microglia and alter their phenotype to the activated state, which in turn may result in an exacerbated inflammatory response and neurodegeneration [33]. Moreover, the CD200–CD200R signalling has been postulated to play a regulatory role by affecting the proliferation and apoptosis of microglial cells [34]. 

Based on the aforementioned data, we hypothesized that MIA might modulate the CX3CL1–CX3CR1 and/or CD200–CD200R pathways, as well as microglia phenotypes in offspring rats subjected either to a prenatal challenge (MIA, as one hit) or to a “two-hit” model of schizophrenia (MIA combined with additional acute immune stimulation), which would lead to the occurrence of distinct behavioural phenotypes in adulthood. 

Therefore, the experiments reported in this paper were designed to verify the impact of MIA on various behavioural parameters (using a range of tests capable of assessing positive, negative and schizoaffective and anxiety-related symptoms) in adult offspring. We also investigated the long-term susceptibility of animals that were prenatally exposed to MIA to an additional LPS challenge in adulthood. In parallel, we examined the gene and protein expression of the CX3CL1–CX3CR1 and CD200–CD200R ligand–receptor axes, as well as microglial pro- and anti-inflammatory phenotypes and the possible microglia priming properties in the context of the “two-hit” hypothesis of schizophrenia.

## 2. Materials and Methods

### 2.1. Animals

Sprague-Dawley rats (Charles River, Sulzfeld, Germany) were maintained under standard conditions: room temperature of 23 °C, 12/12 h light/dark cycle, lights on at 6:00 am, ad libitum access to water and food. After a period of acclimatization, the phase of the oestrous cycle was determined based on the vaginal smears obtained daily from the females. On the pro-oestrus day, the females were placed with males for 12 h and the presence of sperm in vaginal smears was checked the next morning. Pregnant females were randomly divided into two equal groups: (1) control and (2) MIA (*n* = 20 in each group). All procedures were approved by the Animal Care Committee of the Maj Institute of Pharmacology, Polish Academy of Sciences, Cracow and met the criteria of the International Council for Laboratory Animals and Guide for the Care and Use of Laboratory Animals (the consent number: 236/2016; the consent issued exclusively on experiments using male animals). All possible efforts were made to minimize the number of animals used and their suffering. 

### 2.2. Drugs and Treatment

#### 2.2.1. Prenatal Administration of LPS

**Figure 1:**
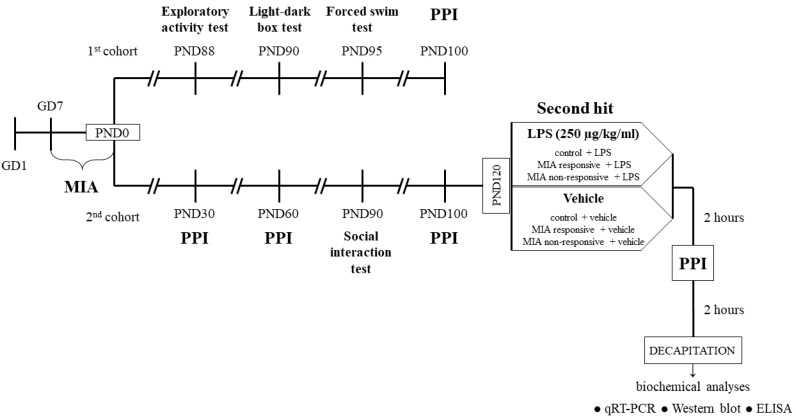
Experimental design. Pregnant dams were exposed to MIA with LPS (2 mg/kg in 1 mL, subcutaneously) beginning on the 7th (GD7) day of pregnancy and continuing every 2nd day until delivery. Control animals were subjected to vehicle (saline) injections on the same schedule. Twenty-one days after birth (PND21), male offspring were separated from dams and housed in groups of 5 per cage under standard conditions. Prior to further experiments, the rats were divided into 2 cohorts. The offspring from the 1st cohort (both the control and MIA groups) were used only for the behavioural examinations, including the exploratory activity test at PND88, light-dark box test at PND90, forced swim test at PND95 and PPI test at PND100. The animals from the 1st cohort were not included in the biochemical analyses. The offspring from the 2nd cohort (both the control and MIA groups) underwent the behavioural examinations in the following order: the PPI test at PND30 and PND60, the social interaction test at PND90 and the PPI test at PND100. At PND120, the animals were divided into 6 groups (control + vehicle, control + LPS, MIA responsive + vehicle, MIA responsive + LPS, MIA non-responsive + vehicle, MIA non-responsive + LPS) and were exposed to the second hit either with LPS (250 µg/kg in 1 mL, intraperitoneally) or vehicle (saline), according to the group assigned. Two hours later, the rats underwent the PPI test and after another 2 h, they were sacrificed by decapitation. The tissues (the frontal cortices and hippocampi) were collected for the biochemical analyses (qRT-PCR, Western blot and ELISA).

LPS (from Escherichia coli 026:B6, Sigma-Aldrich, St. Louis, MO, USA) was dissolved in saline to obtain a concentration of 2 mg/kg in 1 mL and administrated subcutaneously to pregnant rats of the MIA group from the 7th day (GD7) of pregnancy every second day at 10:00 a.m. until delivery [35,36,37]. Control pregnant animals were receiving the corresponding volume (1 mL/kg) of vehicle (saline). Twenty-one days after birth (PND21), male offspring were separated from dams and housed in groups of five per cage under standard conditions. Before further experiments, the rats were divided into two cohorts: the 1st (both the control and MIA groups) was used only for the behavioural examinations, while the 2nd (both the control and MIA groups) underwent behavioural tests and biochemical analyses. The behavioural experiments were performed between 9:00 a.m. and 12:00 a.m. An overview of the experimental design is illustrated in Figure 1. The investigators were not blinded to the experimental conditions. The numbers of animals included in each analysis are presented in the description of the corresponding figure or table.

#### 2.2.2. Additional Immune Activation with LPS in Adulthood

The solution of LPS (from Escherichia coli 026:B6, Sigma-Aldrich, St. Louis, MO, USA) in 1 mL of saline at a concentration of 250 µg/kg was administrated intraperitoneally [38,39] to the male offspring from the control + LPS, MIA responsive + LPS and MIA non-responsive + LPS groups at PND120. The control + vehicle, MIA responsive + vehicle and MIA non-responsive + vehicle groups received an intraperitoneal injection of vehicle (saline) in the corresponding volume (1 mL/kg).

### 2.3. Behavioural Tests

#### 2.3.1. Exploratory Activity Test

The exploratory activity of the 88-day old control and MIA rats was recorded individually for each animal in Opto-Varimex cages (Columbus Instruments, Columbus, OH, USA) linked to an IBM-PC compatible computer (procedure previously described by Basta-Kaim et al. [40]). Each cage (43 × 44 cm) was equipped with 15 infrared emitters located on the horizontal and the vertical axes of the cage and an equivalent number of receivers on the opposite walls of the cage. Exploratory activity was defined as a trespass of three consecutive photo-beams by an animal and it was expressed both as a distance travelled in respective time intervals (5 min each) and as the total distance travelled during 30-min-interval.

#### 2.3.2. Light-Dark Box Test

The light-dark box test was performed based on the procedure reported by Chocyk et al. [41]. To this purpose, we used an apparatus consisting of four cages with the computer-controlled system (TSE Systems, Bad Homburg, Germany). Each experimental box had two compartments: light (covering ¾ of the cage, brightly lit, 100 lx) and dark (covered with a lid), made of clear and black acrylic, respectively. Both sections were permeable to infrared light and were connected by a central gate (10.6 × 10.4 cm). Therefore, the two parts of the cage were freely accessible for the animals to explore. The experimental boxes were located in soundproof, ventilated cabinets, on bases containing integrated infrared sensors along the horizontal and the vertical axes. One hour before the test, the male rats at PND90 from the control and MIA groups were kept in total darkness. The entire experiment was also conducted in a dark room. At the beginning of each testing session, which lasted 10 min, an animal was placed in the one corner of the light compartment, facing away from the gate. The behavioural response during the trials was recorded by Fear Conditioning Software (TSE, Bad Homburg, Germany). Specifically, time spent in each compartment, distance travelled, and average speed were calculated for each animal.

#### 2.3.3. Forced Swim Test

The forced swim test (FST, Porsolt test) was conducted according to the procedure described by Detke et al. [42], which was previously used by our research group [43,44,45,46,47]. The 95-day old male offspring were individually subjected to two trials during which they were forced to swim in a cylinder (50 cm high, 18 cm in diameter) filled with water (23 °C) to a height of 35 cm. The first trial (pretest) lasted 15 min and was intended to accustom the animals to the conditions of the experiment. 24 h after the pretest, the animals were subjected to the second trial (test) which lasted 5 min. During this phase, the total times of immobility, mobility (swimming), and climbing were measured.

#### 2.3.4. Social Interaction Test

The tests investigating social interactions of the animals were performed using the protocol described by Bator et al. [48]. The experiments were conducted in an open field arena (60 × 60 × 30 cm) made of black Plexiglas and dimly illuminated (18 lx) with indirect light. The day before the test, the male offspring at the age of 90 days from the control and MIA groups were transferred to the experimental room and individually adapted to the open field arena for 7 min. Afterwards, half of the rats were marked with potassium permanganate on the rear part of the body. On the test day, two unfamiliar animals (one undyed and one marked) that received identical prenatal treatment were placed in the open field arena. The behaviour of the rats was observed for 10 min by two independent experimenters. The following social behaviours were scored: (1) non-aggressive containing following (rat’s movement towards and following the other rat), sniffing (sniffing parts of the other rat’s body, including an anogenital region), and social grooming (licking and chewing a fur of the other animal), as well as (2) aggressive consisting of attack, fight, and aggressive grooming (aggressive licking and chewing a fur of the other rat). During the test, the time and number of all types of events were measured for each separate animal. Social interactions were expressed as summed scores of the time and the number of aggressive and non-aggressive activities.

#### 2.3.5. Prepulse Inhibition Test (PPI)

The prepulse inhibition test was performed in four time points: when the male offspring were at PND30, PND60, PND100, and PND120, 2 h after the additional injection of LPS. The procedure of the PPI was adopted with some modifications from our previously published studies [35,36,37]. PPI was tested in eight ventilated startle chambers (SR-LAB, San Diego Instruments, California, USA) with a single Plexiglas cylinder (inner diameter of 9 cm) mounted in each of them. A high-frequency loud-speaker inside each chamber produced both a continuous background noise of 65 dB and the various acoustic stimuli. The average startle amplitudes (AVG) were detected for each animal by a piezoelectric accelerometer and then digitized and used for subsequent analyses. Before the experiments, each chamber was individually calibrated by the external sensor to display a similar readout of the reference stimulus. The AVGs were measured during the 200-millisecond-recording window. After 5 min of habituation with the background noise, four types of acoustic stimuli were used in random order. Each trial consisted of either a single pulse alone [intensity: 120 dB, duration: 40 milliseconds, (P)], or a pulse preceded by a prepulse at one out of three intensities (70, 75, and 80 dB; duration: 20 milliseconds; (PP)) applied 80 milliseconds before a pulse. During each experimental session, 20 trials of each type were presented with an interstimulus interval of 20 s. The AVGs were recorded and the percentage of PPI (%PPI) induced by each prepulse intensity was calculated as %PPI = [(P − PP)/P] × 100%.

At PND100, the offspring from the MIA group were divided into two categories: MIA responsive (with the deficit in PPI) and MIA non-responsive (without the deficit). The subcategorization was done based on the PPI results calculated with the AVGs for the 75 dB prepulse. First, the mean response in PPI at 75 dB prepulse was calculated for the control group. Then, the MIA offspring were divided in such a way that all animals with %PPI lower than the average response of the control rats were categorized as “MIA responsive” and all animals with %PPI higher than the mean for the control group were assigned to “MIA non-responsive” group. Categories obtained for 75 dB prepulse were maintained for the remaining prepulse intensities (70 and 80 dB).

### 2.4. Biochemical Analyses

#### 2.4.1. Tissues Collection

The tissues collection from the adult animals of all groups was done 4 h after the additional injection of LPS. The frontal cortices (Cx) and hippocampi (Hp) were dissected on an ice-cold glass plate and then stored at −80 °C before being used for further treatment. 

#### 2.4.2. Tissues Preparation

Collected tissues (one frontal cortex and one hippocampus) from all groups were homogenized with a RIPA lysis buffer with protease inhibitor cocktail, phosphatase inhibitor cocktail, 1 mM sodium orthovanadate and 1 mM phenylmethanesulfonyl fluoride (all from Sigma-Aldrich, St. Louis, MO, USA) by the Tissue Lyser II (Qiagen Inc, Valencia, CA, USA). The protein concentrations in the analysed samples were determined using the BCA Protein Assay Kit (Sigma-Aldrich, St. Louis, MO, USA) with bovine serum albumin as a standard and measured at a wavelength of 562 nm at Tecan Infinite 200 Pro spectrophotometer (Tecan, Mannedorf, Germany). The samples were further used for biochemical analyses with enzyme-linked immunosorbent assay (ELISA) and Western blot techniques.

#### 2.4.3. Quantitative Real-Time Polymerase Chain Reaction (qRT-PCR)

Total RNA was extracted from the other part of the frontal cortices and hippocampi of 120-day old male rats using the GeneMATRIX Universal RNA Purification Kit (EURx, Gdańsk, Poland) according to the manufacturer’s instructions. The samples were homogenized in an appropriate volume of the lysis buffer supplied with the kit by the Tissue Lyser II (Qiagen Inc, Valencia, CA, USA), and isolation of total RNA was performed with strict adherence to the manufacturer’s instructions. Immediately after extraction, the concentration of RNA was determined by a NanoDrop Spectrophotometer (ND/1000 UV/Vis, Thermo Fisher NanoDrop, Waltham, MA, USA). The synthesis of the complementary DNA (cDNA), via reverse transcription, from equal amounts of RNA (1 µg) was performed using NG dART RT kit (EURx, Gdańsk, Poland). The cDNA was amplified with the usage of FastStart Universal Probe Master (Rox) kit (Roche, Basel, Switzerland), TaqMan probes (Life Technologies, Carlsbad, CA, USA) for the genes: *Cx3cl1* (Rn00593186_m1), *Cx3cr1* (Rn00591798_m1), *Cd200* (Rn01646320_m1), *Cd200r* (Rn00576646_m1), *MhcII* (Rn01424725_m1), *Cd40* (Rn01423583_m1), *iNos* (Rn00561646_m1), *Il-1β* (Rn00580432_m1), *Tnf-α* (Rn00562055_m1), *Il-6* (Rn01410330_m1), *Arg1* (Rn00691090_m1), *Igf-1* (Rn00710306_m1), *Tgf-β* (Rn00572010_m1), *Il-4* (Rn01456866_m1) and, as the reference, *B2m* (Rn00560865_m1), or *Hprt* (Rn01527840_m1). The PCR products were generated in the mixtures consisting of cDNA used as the PCR template (1 µL), TaqMan forward and reverse primers (1 µL), 1× FastStart Universal Probe Master (Rox) mix containing 250 nM of hydrolysis probe labelled with the fluorescent reporter dye (fluorescein (FAM)) at the 5′-end and a quenching dye at the 3′-end (10 µL), and finally the remainder of PCR grade distilled water to a total volume of 20 µl. The thermocycling conditions contained an initial denaturation at 95 °C for 10 min followed by 45 cycles of denaturation at 95 °C for 15 s, annealing at 60 °C for 1 min, and extension at 50 °C for 2 min. The threshold value (*C*t) for each sample was set in the exponential phase of PCR, and the ∆∆*C*t method was used for the data analysis.

#### 2.4.4. Enzyme-Linked Immunosorbent Assay (ELISA)

The protein levels of CX3CL1 (Cloud-Clone Corp., Katy, TX, USA), CX3CR1, CD200, CD200R, IL-6, and IL-4 (all from Cusabio, Houston, TX, USA) in the frontal cortices and hippocampi of the male rats were measured using commercially available ELISA kits. The procedures were performed in accordance with the manufacturer’s instructions and the minimum detectable doses were: CX3CL1: 0.055 ng/mL, CX3CR1: 5.8 pg/mL, CD200: 11.75 pg/mL, CD200R: 4.67 pg/mL, IL-6: 0.78 pg/mL, IL-4: 3.9 pg/mL. Intra- and inter-assay precision values were: CX3CL1: <10%, <12%, CX3CR1, CD200, CD200R, IL-6, IL-4: <8%, and <10%, respectively. 

#### 2.4.5. Western Blot

The samples containing equal amounts of protein (10 µg) were mixed with Laemmli sample buffer (Bio-Rad, Hercules, CA, USA) in a 4:1 ratio (*v*/*v*) and heated at 95 °C for 8 min using Eppendorf Thermomixer comfort (Sigma-Aldrich, St. Louis, MO, USA). Proteins were electrophoretically separated by SDS–PAGE (4–20% gel; Bio-Rad, Hercules, CA, USA) under constant voltage (200 V) and then transferred to PVDF membranes (Sigma-Aldrich, St. Louis, MO, USA) in Trans-Blot Turbo (Bio-Rad, Hercules, CA, USA). The blots were cut into two parts, rinsed 3 × 10 min with tris-buffered saline solution (TBS), and blocked in 5% bovine serum albumin dissolved in TBS with 0.1% Tween 20 (TBST) (both from Sigma-Aldrich, St. Louis, MO, USA) for 1 h at room temperature (RT). After washing 3 × 10 min in TBST, the membranes were incubated overnight at 4 °C with the anti-IBA1 (NBP2-19019, 1:500, Novus Biologicals, Centennial, CO, USA) or anti-β-actin (A5441, 1:10000, Sigma-Aldrich, St. Louis, MO, USA) antibody diluted in a SignalBoost Immunoreaction Enhancer Kit (Millipore, Warsaw, Poland). The next day the blots were 3 × 10 min rinsed with TBST and incubated with appropriate peroxidase-conjugated secondary antibody: goat anti-rabbit IgG (PI-1000, 1:2500) or goat anti-mouse IgG (BA-9200, 1:4000) (both from Vector Laboratories, Peterborough, UK) for 1 h at RT. After washing 3 × 10 min in TBST, the immune complexes were detected using Pierce™ ECL Western Blotting Substrate (Thermo Fisher, Pierce Biotechnology, Carlsbad, CA, USA) and visualized using a Fujifilm LAS-1000 System (Fuji Film, Tokyo, Japan). The relative levels of immunoreactivity were densitometrically quantified using Fujifilm Multi Gauge software (Fuji Film, Tokyo, Japan).

### 2.5. Statistical Data Analysis

Statistical analysis of the data was performed using the Statistica 13.0 Software (StatSoft, Palo Alto, CA, USA). The results from behavioural examinations are demonstrated as the means ± standard errors of the mean (SEM). The data from qRT-PCR studies are displayed as the average folds ± SEM, those from ELISA experiments are presented as the means ± SEM, and from Western blot analyses—as IBA1/β-actin ratio ± SEM. Comparisons of variables between groups were analysed as follows: for exploratory activity, light-dark box test, forced swim test, social interaction test, and the PPI at PND30, PND60, and PND100 with the Student’s *t*-test; for the PPI after the additional injection of LPS and biochemical experiments using qRT-PCR and ELISA with planned comparisons in one-way ANOVA (contrast analysis); for the Western blot study with Kruskal–Wallis test. The results were considered statistically significant when the *p*-value was lower than 0.05. All precise data presented as the means ± SEM are provided in the Supplementary Materials. All graphs were prepared with the usage of GraphPad Prism 7 Software (San Diego, CA, USA). 

## 3. Results

### 3.1. Exploratory Activity

**Figure 2:**
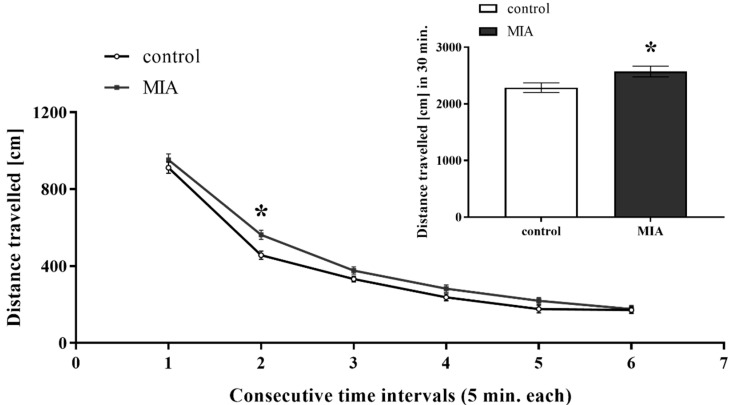
The effect of MIA on the exploratory activity of adult male Sprague-Dawley offspring. The exploratory activity is expressed as a distance travelled (in cm) in respective time intervals (5 min each) and the total distance travelled during 30-min-interval (showed on the inset). *n* = 42 in the control group, *n* = 69 in the MIA group. The results are presented as the means ± SEM. * *p* < 0.05 vs. control group.

Exploratory behaviour is induced by novel stimuli and allows animals to collect information about unfamiliar parts of an environment [49]. In our study, adult male offspring from the MIA group were more active than the control rats, as evidenced by an increase in the total distance travelled (*p* = 0.0389) (Figure 2). The statistical analysis showed that the exploration of the MIA animals was particularly enhanced in the second interval of the experiment (*p* = 0.0029) (Figure 2). Hence, MIA modulated the novelty-related behaviour of the offspring.

### 3.2. Light-Dark Box Test

**Figure 3:**
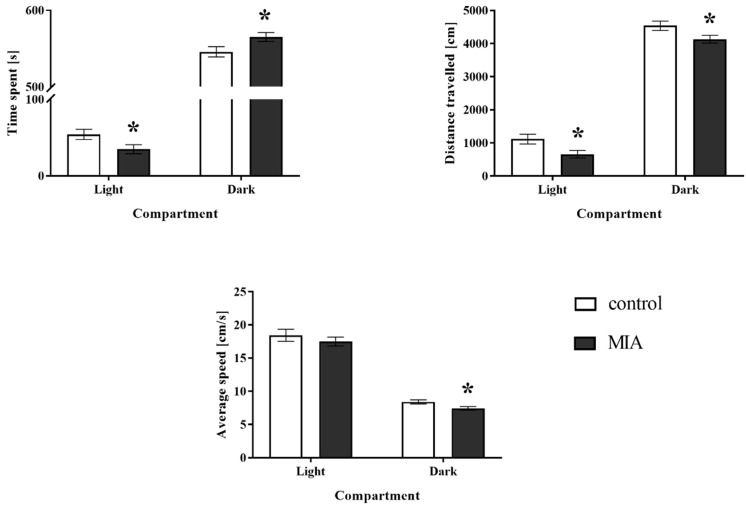
The effect of MIA on anxiety-like behaviours of adult male Sprague-Dawley offspring, measured in the light-dark box test. *n* = 42 in the control group, *n* = 69 in the MIA group. The results are presented as the means ± SEM. * *p* < 0.05 vs. control group.

We performed light-dark box test to examine the effect of MIA on anxiety-like behaviour in adult rat offspring (Figure 3). The animals prenatally treated with LPS were characterized by a decrease in the time spent (*p* = 0.0401) and distance travelled (*p* = 0.0162) in the light compartment when compared to the control rats. For the dark part of the apparatus, we observed that all three parameters measured were significantly influenced by MIA: time spent (*p* = 0.0401), distance travelled (*p* = 0.0299), and average speed (*p* = 0.0213) (Figure 3). In summary, MIA affected the anxiety level in the adult offspring.

### 3.3. Forced Swim Test

**Figure 4:**
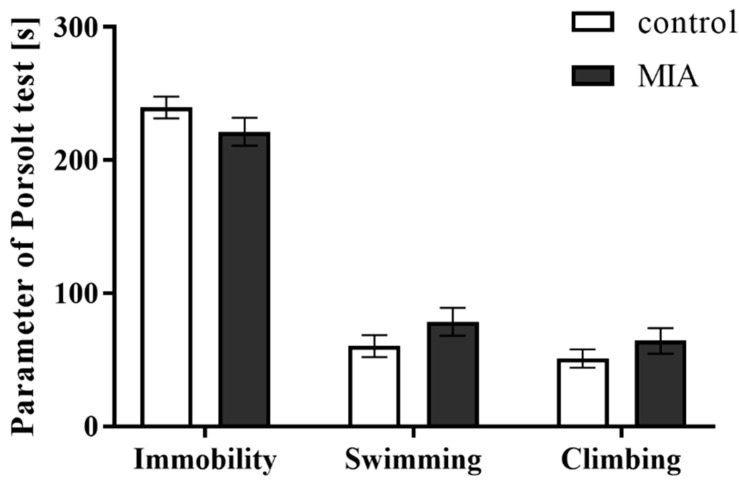
The effect of MIA on depressive-like behaviours of adult male Sprague-Dawley offspring, measured in the forced swim test. *n* = 20 in each group. Immobility, swimming and climbing times (in seconds) are presented as the means ± SEM.

Some behavioural deficits are common both for schizophrenic and schizoaffective patients [50,51]. In the present study, we used the FST to examine whether the adult animals from the MIA group displayed depressive-like behaviour that is a characteristic of affective disorders. The obtained results (Figure 4) revealed that MIA did not affect any analysed parameter (immobility, swimming, and climbing). Therefore, MIA did not induce depressive-like changes in adult male offspring.

### 3.4. Social Interaction Test

Social deficits substantially affect the functioning of patients with schizophrenia and, accordingly, are one of the negative symptoms of the disease [52]. Moreover, some studies reported an inverse correlation between social interaction and anxiety levels [53] as well as the bidirectional relationship between social behaviour and immune signalling [54]. To our surprise, the treatment of pregnant females with LPS did not induce disturbances either in the time or the number of non-aggressive and aggressive behaviours in the adult male offspring (Table 1).

### 3.5. Prepulse Inhibition of the Acoustic Startle Response

The PPI paradigm is commonly used to evaluate sensorimotor gating, which is disturbed both in patients [55,56,57] and animal models of the disease [58,59]. In the first set of experiments, we examined the effect of MIA on the PPI response of male rat offspring at two time points: at PND30 and PND60. As shown in Table 2, MIA led to the raised PPI for the 75 dB prepulse level (*p* = 0.0157) in animals at PND30. At PND60, the prenatal administration of LPS to pregnant dams did not disrupt the PPI in offspring (Table 2). 

**Figure 5:**
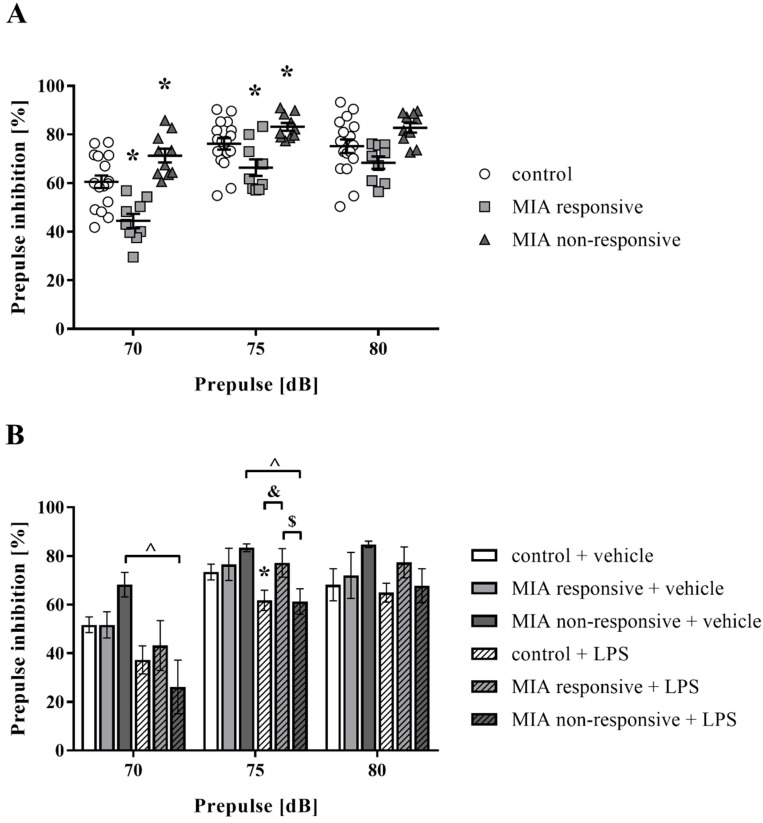
(**A**) The PPI test at PND100 revealed two distinct behavioural phenotypes: MIA responsive (with the deficit in PPI) and MIA non-responsive (without the deficit) in male Sprague-Dawley offspring exposed to MIA. *n* = 17 in the control group, *n* = 9 in the MIA responsive group, *n* = 10 in the MIA non-responsive group. * *p* < 0.05 vs. control group. The results are presented as the individual data points of the percentage of PPI (%PPI) induced by each prepulse intensity with the means ± SEM. Data were calculated based on the average startle amplitudes (AVGs). (**B**) At PND120, the animals were additionally subjected to the acute challenge with LPS and 2 h later, the PPI was evaluated again. *n* = 4–10. * *p* < 0.05 vs. control + vehicle, ^ *p* < 0.05 vs. MIA non-responsive + vehicle, $ *p* < 0.05 vs. MIA responsive + LPS, & *p* < 0.05 vs. control + LPS. The results are presented as the means of the percentage of PPI (%PPI) induced by each prepulse intensity ± SEM. Data were calculated based on the average startle amplitudes (AVGs).

Interestingly, at PND100, we identified two response patterns in the obtained results. Based on this observation, the rats were divided into two categories: MIA responsive (with the deficit in PPI) and MIA non-responsive (without the deficit) (Figure 5A). The MIA responsive group displayed significant inhibition of sensorimotor gating compared to the control offspring for the 70 dB (*p* = 0.0008) and 75 dB (*p* = 0.0252) prepulse intensities. The MIA non-responsive rats were characterized by an increase in the PPI compared to the control offspring for the 70 dB (*p* = 0.0126) and 75 dB (*p* = 0.0475) prepulse levels.

In the next step, we implemented the “two-hit” hypothesis of schizophrenia and examined whether the additional acute challenge with LPS in adulthood further altered the PPI in the male offspring of dams after MIA (Figure 5B). The additional immune stimulation did not aggravate the disruption of sensorimotor gating in the group of MIA responsive animals. The outcome after the “second hit” for those rats was significantly different from the control (*p* = 0.0182) and the MIA non-responsive (*p* = 0.0335) groups for a prepulse stimulus of 75 dB. Importantly, the MIA non-responsive offspring exhibited a decrease in the PPI for the 70 dB (*p* = 0.0005) and 75 dB (*p* = 0.0042) prepulse levels, when treated with LPS in adulthood. Additionally, the acute administration of LPS significantly reduced the PPI in the control group for the 75 dB (*p* = 0.0440) prepulse intensity (Figure 5B). 

### 3.6. The mRNA Expression of the Cx3cl1, Cx3cr1, Cd200 and Cd200r in the Frontal Cortices and Hippocampi of Adult Male Offspring

**Figure 6:**
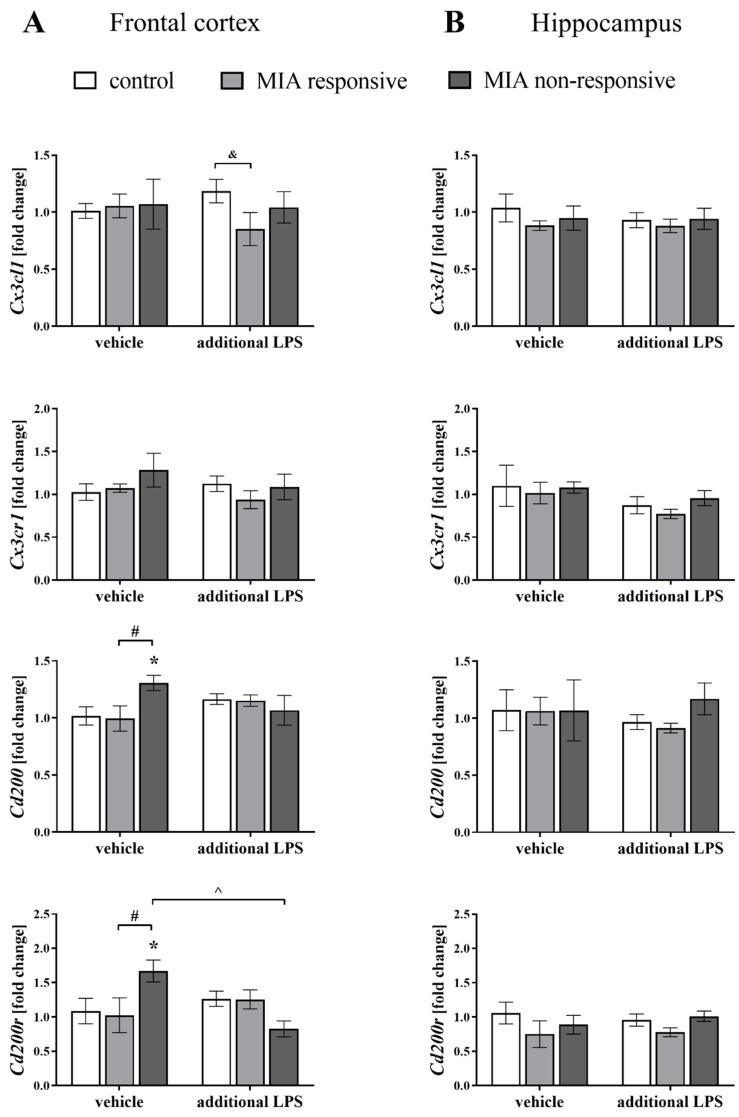
The effect of MIA and the additional acute challenge with LPS on the gene expression of *Cx3cl1*, *Cx3cr1*, *Cd200* and *Cd200r* in the frontal cortices (**A**) and hippocampi (**B**) of male Sprague-Dawley offspring at PND120. The mRNA levels were measured using qRT-PCR with *n* = 3–10 in each group. The results are presented as the average fold change ± SEM. * *p* < 0.05 vs. control + vehicle, # *p* < 0.05 vs. MIA responsive + vehicle, ^ *p* < 0.05 vs. MIA non-responsive + vehicle, & *p* < 0.05 vs. control + LPS.

In the first set of biochemical experiments, we determined the mRNA expression of neuronal ligands (*Cx3cl1*, *Cd200*) and their corresponding microglial receptors (*Cx3cr1*, *Cd200r*) in the frontal cortices and hippocampi of male offspring after MIA and the additional acute systemic injection of LPS in adulthood using qRT-PCR (Figure 6). 

The most striking changes were observed in the expression of the *Cd200-Cd200r* axis in the frontal cortices of the MIA non-responsive animals. In more detail, MIA increased the cortical *Cd200* level in the MIA non-responsive offspring when compared to the control (*p* = 0.0313) and the MIA responsive (*p* = 0.0345) animals. For the MIA non-responsive rats, we also observed an elevation of the *Cd200r* level in the frontal cortex when compared to the control (*p* = 0.0329) and the MIA responsive (*p* = 0.0340) offspring, and detected a significant reduction in the *Cd200r* expression after the additional treatment with LPS (*p* = 0.0050) (Figure 6A).

Contrast analysis showed a significant decrease in the *Cx3cl1* level in the frontal cortex of the MIA responsive rats after the “second hit” with LPS when compared to the control group (*p* = 0.0456). The hippocampal gene expression of all analysed factors was not affected either by the prenatal treatment or the acute stimulation with LPS (Figure 6B), which clearly showed that MIA and the additional LPS treatment only affected ligand–receptor expression in the frontal cortex.

### 3.7. Levels of the CX3CL1, CX3CR1, CD200 and CD200R Proteins in the Frontal Cortices and Hippocampi of Adult Male Offspring

**Figure 7:**
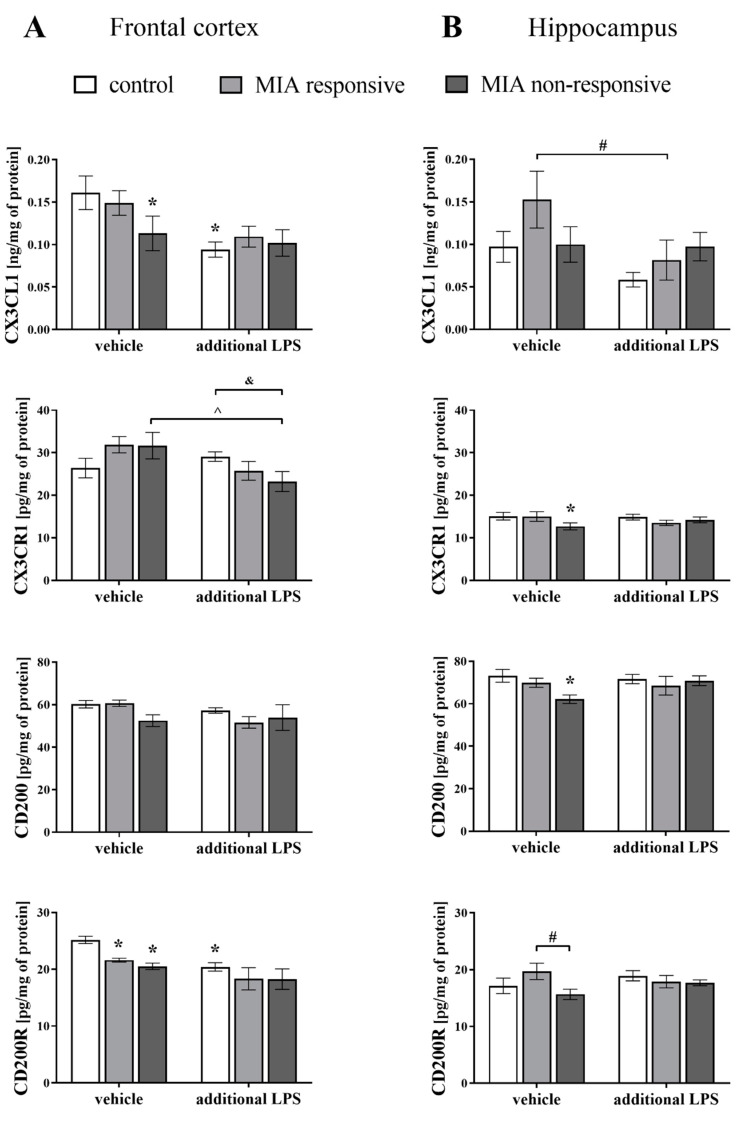
The effect of MIA and the additional acute challenge with LPS on the protein levels of CX3CL1, CX3CR1, CD200 and CD200R in the frontal cortices (**A**) and hippocampi (**B**) of male Sprague-Dawley offspring at PND120. *n* = 4–10 in each group. The results are presented as the means ± SEM. * *p* < 0.05 vs. control + vehicle, # *p* < 0.05 vs. MIA responsive + vehicle, ^ *p* < 0.05 vs. MIA non-responsive + vehicle, & *p* < 0.05 vs. control + LPS.

Considering the alterations in mRNA expression, in the next step of the study, we determined the protein levels of the systems controlling neuron–microglia interactions in the brains of adult rats after MIA and the acute immune stimulation with LPS (Figure 7).

Interestingly, similar to the gene expression patterns, the greatest MIA-evoked changes were observed in the frontal cortex of the MIA non-responsive offspring. The cortical CX3CL1 levels were lowered in the MIA non-responsive (*p* = 0.0382) and the control (*p* = 0.0011) groups after the “second hit” with LPS.

Consistent with these results, the acute injection of LPS decreased the level of CX3CR1 in the frontal cortices of the MIA non-responsive animals (*p* = 0.0137), and the effect of the treatment was more substantial than in the control offspring (*p* = 0.0440). 

Regarding the levels of CD200R, the impact of MIA was pronounced for both the MIA responsive (*p* = 0.0423) and the MIA non-responsive (*p* = 0.0059) groups. Similarly, a decline in the CD200R level was detected for the control animals that were additionally treated with LPS in adulthood (*p* = 0.0011) (Figure 7A). 

An analysis of the hippocampal homogenates of the MIA non-responsive offspring revealed reduced levels of CX3CR1 (*p* = 0.0491) and CD200 (*p* = 0.0111) when compared to the control animals, and CD200R (*p* = 0.0348) when compared to the MIA responsive rats (Figure 7B). 

The additional injection of LPS resulted in a decrease in the CX3CL1 level in the hippocampus of the MIA responsive group (*p* = 0.0245).

### 3.8. The IBA1 Levels in the Frontal Cortices and Hippocampi of Adult Male Offspring

**Figure 8:**
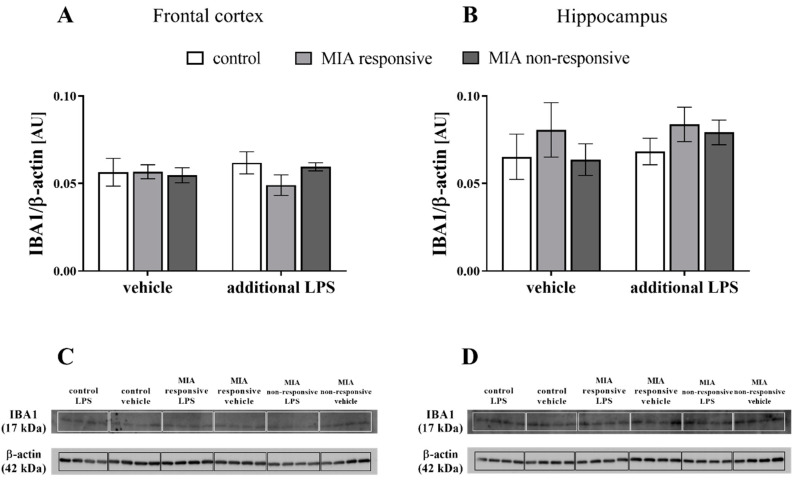
The effect of MIA and the additional acute challenge with LPS on the protein level of IBA1 in the frontal cortices (**A**) and hippocampi (**B**) of male Sprague-Dawley offspring at PND120. *n* = 4 in each group. The results are presented as IBA1/β-actin ratio ± SEM. (**C**,**D**) The representative immunoblots for each group.

After identifying the changes in microglial CX3CR1 and CD200R levels, we assessed IBA1 levels in the frontal cortices and hippocampi of the adult animals after MIA and the additional challenge with LPS (Figure 8). The Western blot analysis showed no significant differences in either the cortical or hippocampal levels of IBA in the offspring from any examined group.

### 3.9. The mRNA Expression of the Microglial Markers in the Frontal Cortices and Hippocampi of Adult Male Offspring

**Figure 9:**
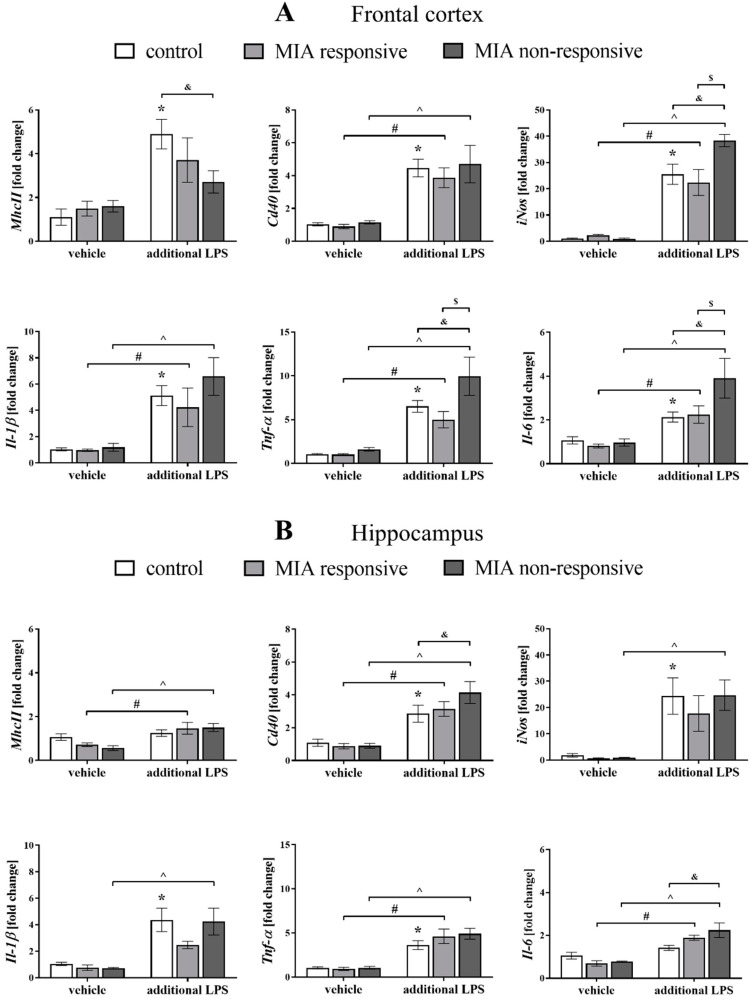
The effect of MIA and the additional acute challenge with LPS on the gene expression of the pro-inflammatory microglial markers: *MhcII*, *Cd40*, *iNos*, *Il-1β*, *Tnf-α* and *Il-6* in the frontal cortices (**A**) and hippocampi (**B**) of male Sprague-Dawley offspring at PND120. The mRNA levels were measured using qRT-PCR with *n* = up to 10 in each group. The results are presented as the average fold change ± SEM. * *p* < 0.05 vs. control + vehicle, # *p* < 0.05 vs. MIA responsive + vehicle, ^ *p* < 0.05 vs. MIA non-responsive + vehicle, $ *p* < 0.05 vs. MIA responsive + LPS, & *p* < 0.05 vs. control + LPS.

**Figure 10:**
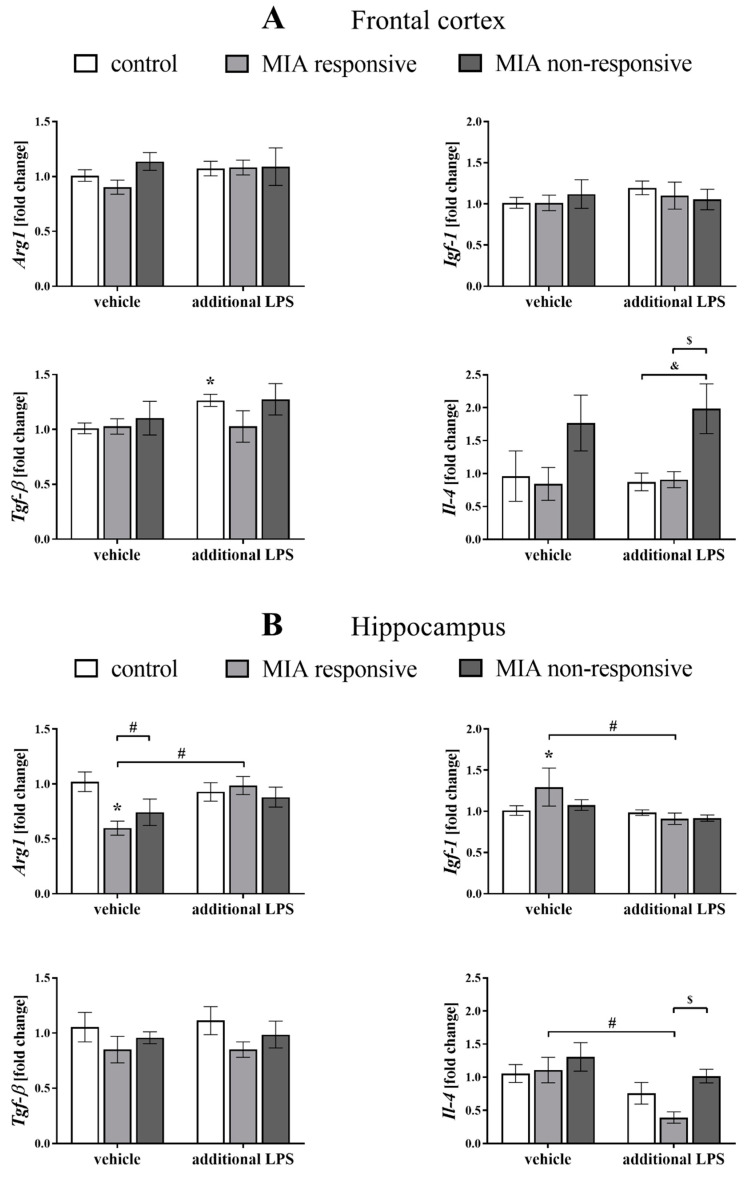
The effect of MIA and the additional acute challenge with LPS on the gene expression of the anti-inflammatory microglial markers: *Arg1*, *Igf-1*, *Tgf-β* and *Il-4* in the frontal cortices (**A**) and hippocampi (**B**) of male Sprague-Dawley offspring at PND120. The mRNA levels were measured using qRT-PCR with *n* = up to 10 in each group. The results are presented as the average fold change ± SEM. * *p* < 0.05 vs. control + vehicle, # *p* < 0.05 vs. MIA responsive + vehicle, $ *p* < 0.05 vs. MIA responsive + LPS, & *p* < 0.05 vs. control + LPS.

Because the data suggested that microglial cells might be intimately involved in the pathogenesis of schizophrenia [60,61], we explored potential alterations in the pro- (*MhcII*, *Cd40*, *iNos*, *Il-1β*, *Tnf-α* and *Il-6*) and anti-inflammatory (*Arg1*, *Igf-1*, *Tgf-β* and *Il-4*) factors that are also considered microglial markers (Figure 9 and Figure 10).

Regarding the results obtained from the frontal cortex, we did not observe an effect of MIA on the expression of any of the analysed pro-inflammatory microglial markers in the MIA responsive or non-responsive offspring. 

However, after the “second hit” in the MIA responsive animals, the mRNA levels of *Cd40* (*p* = 0.0016), *iNos* (*p* = 0.0057), *Il-1β* (*p* = 0.0239), *Tnf-α* (*p* = 0.0212) and *Il-6* (*p* = 0.0172) were significantly upregulated (Figure 9A). 

The impact of the additional injection of LPS was also observed in the MIA non-responsive offspring, where an elevation of the cortical expression of *Cd40* (*p* = 0.0011), *iNos* (*p* < 0.0001), *Il-1β* (*p* = 0.0012), *Tnf-α* (*p* < 0.0001) and *Il-6* (*p* = 0.0001) was detected. The most intriguing observation was that the changes for the MIA non-responsive rats that received the LPS in adulthood were more distinct than in the MIA responsive group at the levels of *iNos* (*p* = 0.0019), *Tnf-α* (*p* = 0.0036) and *Il-6* (*p* = 0.0068), as well as those in the control animals for *iNos* (*p* = 0.0067), *Tnf-α* (*p* = 0.0129) and *Il-6* (*p* = 0.0020). The cortical *MhcII* level in the MIA non-responsive offspring exposed to the additional stimulation with LPS was lower than in the control rats (*p* = 0.0324) (Figure 9A). 

Additionally, the acute treatment with LPS increased the expression of all tested markers of the pro-inflammatory microglial phenotype in the frontal cortices of the control animals: *MhcII* (*p* = 0.0001), *Cd40* (*p* < 0.0001), *iNos* (*p* < 0.0001), *Il-1β* (*p* = 0.0003), *Tnf-α* (*p* = 0.0001) and *Il-6* (*p* = 0.0216) (Figure 9A). 

Among the tested markers of the anti-inflammatory phenotype, we observed that in the frontal cortices of the MIA non-responsive rats after the “second hit” with LPS, the mRNA level of *Il-4* was higher than in the control (*p* = 0.0047) and the MIA responsive (*p* = 0.0127) offspring (Figure 10A). Moreover, the acute injection with LPS increased *Tgf-β* expression in the control group (*p* = 0.0157).

Analyses of samples obtained from the hippocampi of the MIA responsive offspring revealed that the acute stimulation with LPS resulted in an increase of the levels of *MhcII* (*p* = 0.0229), *Cd40* (*p* = 0.0034), *Tnf-α* (*p* < 0.0001) and *Il-6* (*p* = 0.0001). A similar effect was demonstrated for the MIA non-responsive animals, as evidenced by the raised hippocampal expression of *MhcII* (*p* = 0.0017), *Cd40* (*p* < 0.0001), *iNos* (*p* = 0.0014), *Il-1β* (*p* = 0.0010), *Tnf-α* (*p* < 0.0001), and *Il-6* (*p* < 0.0001) after the “second hit” with LPS. Simultaneously, the mRNA levels of *Cd40* (*p* = 0.0413) and *Il-6* (*p* = 0.0017) in the hippocampi of the MIA non-responsive rats after the additional injection of LPS were significantly higher than in the control offspring. Increased levels of *Cd40* (*p* = 0.0045), *iNos* (*p* = 0.0008), *Il-1β* (*p* = 0.0005), and *Tnf-α* (*p* = 0.0003) were also observed in the hippocampi of the control animals after the stimulation with LPS in adulthood (Figure 9A). 

As in the case of the frontal cortex, we observed a less profound impact of MIA and/or the acute LPS treatment on the expression of anti-inflammatory microglial markers in the hippocampus. A statistical analysis of the levels of the anti-inflammatory factors in the hippocampi of the MIA responsive rats showed that MIA affected the mRNA expression of *Arg1* (*p* = 0.0058) and *Igf-1* (*p* = 0.0236). The acute treatment with LPS also altered the expression of *Arg1* (*p* = 0.0132) and *Igf-1* (*p* = 0.0052). The hippocampal *Arg1* level in the MIA responsive group was significantly lower than in the MIA non-responsive offspring (*p* = 0.0452). The injection of LPS in adulthood decreased the *Il-4* expression in the hippocampi of the MIA responsive animals (*p* = 0.0123), and that change was significantly different from the level of this cytokine in the MIA non-responsive rats (*p* = 0.0205) (Figure 10B).

### 3.10. Levels of the IL-6 and IL-4 Proteins in the Frontal Cortices and Hippocampi of Adult Male Offspring

**Figure 11:**
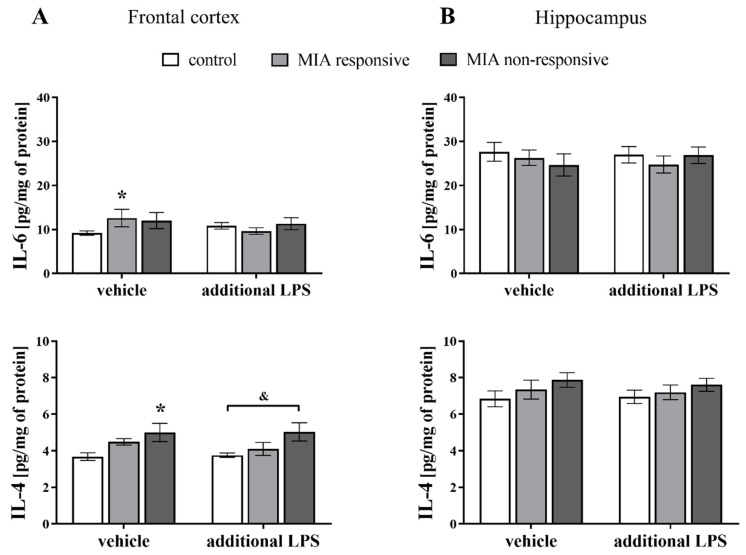
The effect of MIA and the additional acute challenge with LPS on the protein levels of the pro-inflammatory cytokine IL-6 and the anti-inflammatory cytokine IL-4 in the frontal cortices (**A**) and hippocampi (**B**) of male Sprague-Dawley offspring at PND120. *n* = 4–10 in each group. The results are presented as the means ± SEM. * *p* < 0.05 vs. control + vehicle, & *p* < 0.05 vs. control + LPS.

In the next set of experiments, we examined whether MIA and the acute systemic treatment with LPS in adulthood affected the cytokine profile in the brains of male rats (Figure 11). We focused on two factors: the pro-inflammatory IL-6, which has a potential role in the induction of schizophrenia-like behavioural disturbances [62,63], and the anti-inflammatory IL-4, which is the main cytokine regulating the CD200–CD200R axis [64,65]. Regarding the data for the frontal cortex, there was a significant increase in the levels of IL-6 in the MIA responsive animals (*p* = 0.0457) and IL-4 in the MIA non-responsive rats (*p* = 0.0029) when compared to the control group. The MIA non-responsive offspring were more susceptible to the additional injection with LPS (*p* = 0.0042) than the control group in terms of the IL-4 level in the frontal cortex (Figure 11A). The ELISA results revealed no changes in the levels of the IL-6 or IL-4 proteins in the hippocampi of the offspring from any of the investigated groups (Figure 11B).

## 4. Discussion

Prenatal MIA, which was generated by LPS exposure in the last two weeks of pregnancy, induced schizophrenia-like behavioural changes in adult male Sprague-Dawley offspring, such as impairments in the exploratory activity and the presence of anxiety behaviours. The sensorimotor gating deficit was age-dependent and present only in part of the animals, leading to the occurrence of two behavioural phenotypes: responsive (with the deficit in PPI) and non-responsive (without the deficit). We are the first to report that MIA disrupted the CD200–CD200R system in the frontal cortex of adult Sprague-Dawley offspring (mostly from the non-responsive group), while the changes in the CX3CL1–CX3CR1 proteins were less evident. MIA did not change the pro- and anti-inflammatory microglial phenotypes either in the frontal cortex or hippocampus at the mRNA level, while it markedly increased the cortical IL-6 release in the responsive rats and IL-4 release in the non-responsive offspring. Importantly, the “second hit” in the form of systemic acute LPS treatment in adulthood only generated disturbances at the behavioural and biochemical levels in the non-responsive adult animals. Those offspring were characterized both by disrupted PPI and “priming” microglia, as evidenced by the upregulation of pro-inflammatory factors in both the frontal cortex and hippocampus.

In the present study, we observed increased exploratory behaviour in adult animals exposed to MIA, that was evoked by novel stimuli and related to the collection of information about unfamiliar parts of the environment. These results are consistent with observations of Wistar rats [35,36,40] and reports showing hyperactivity in response to a novel environment in various neurodevelopmental animal models of schizophrenia [66,67]. These findings are particularly relevant in light of the data classifying the exploratory activity in animals as a manifestation of positive symptoms that are comparable with the psychomotor agitation present in some schizophrenic patients [68]. 

In contrast, anxiety and social withdrawal are described as negative symptoms of schizophrenia [52,69]. In our study, the MIA-exposed rats spent more time in the dark (protected) compartment of the apparatus than control animals. This result, showing an aversion to the unprotected (light) section of the box, may indicate the presence of anxiety-like behaviour in the MIA-subjected rats. Concurrently, the shorter distance travelled in the dark part might result from the conflict between the exploratory drive and risk avoidance [70]. Consistent with our data, rats prenatally treated with LPS displayed more anxiety-like behaviours and enhanced stress responses [71]. Also, the offspring exposed to intrauterine inflammation [72] and vaginitis [73] before birth, which are both induced by LPS injection, manifested anxiety-related behaviours. Nevertheless, different species or strains, as well as diverse test conditions, exert crucial effects on the outcome of animal examinations of anxiety [74].

Regarding the test of social interactions, we did not note an impact of MIA on the number of either social or aggressive events in adulthood. This finding contrasts with the data obtained for Wistar rats, described previously by our team [40] and by Kirsten et al. [75], suggesting that social interactions strongly depend on an interaction between the genetic background and experimental design [53,76]. In the present study, we applied a different protocol [48] than the procedure based on the assessment of social interaction in the resident–intruder paradigm which was used previously [40]. 

In patients, schizophrenia symptoms often coincide with mood disturbances, constituting schizoaffective disorder [77]. Accordingly, we employed the FST, a commonly used procedure to evaluate depressive-like behaviours in rodents [42,78]. None of the analysed parameters (immobility, swimming and climbing) were affected by MIA during pregnancy, suggesting that adult male offspring displayed schizophrenia symptoms rather than a schizoaffective disorder. Several earlier animal studies have shown the opposite effect of prenatal MIA on depression-like behaviours in offspring compared to the data presented here [79,80,81]. We did not find a negative correlation between novelty-related behaviour and immobility or climbing time in the FST, which has been postulated by some authors [82]. However, the majority of observations differing from our results were derived from experiments utilizing other models of schizophrenia that all used different applied conditions, including the dose, timing and route of an endotoxin injection to pregnant dams. The differences may also result from the strain of the animals used in our research. Notably, Rybnikova et al. [83] performed comparative behavioural studies and found that among the various strains of rats, Sprague-Dawley rats were characterized by the highest exploratory activity but the lowest index of depressive-like behaviour. It is also important to note that exploratory, anxiety-, and depression-related behaviours are governed by various mechanisms. The fact that those behaviours can be mediated by different brain structures (e.g., hippocampus, frontal cortex, etc.) and inputs from more regions [84,85,86,87,88] should also be taken into account.

Patients with schizophrenia typically experience a combination of symptoms, yet, do not manifest every possible sign of the disease. Thus, animal models of schizophrenia do not serve as the complete animal equivalent of the human disease and do not necessarily exhibit malfunctions in all behaviours relevant to this condition [89]. We expected that the MIA model used in this study would recapitulate some features of schizophrenia. Therefore, we assessed sensorimotor gating by measuring alterations in PPI, which dictates a particular “endophenotype” of the disease both in humans and rodents [90,91]. Sensorimotor gating is a process that allows an animal to allocate attentional resources to more salient stimuli in the environment [90]. The functional basis of PPI is regulated by the brainstem, but it is highly modulated by cerebral [92] and hippocampal [93] inputs as well as dopamine [94] and serotonin [95,96] transmission. Here, we provide evidence that MIA in Sprague-Dawley offspring did not interfere with sensorimotor gating in adolescence. Contrary, in adulthood, the changes in the PPI test were manifested as two behavioural phenotypes: responsive (with the deficit) and non-responsive, in which the PPI deficit was absent. Behavioural diversity in response to MIA makes this model a better approximation of patients with schizophrenia, because only 1% of people develop symptoms, despite the epidemiological evidence of a broader role for prenatal exposure to adverse effects of various immune stimuli. Our present results contradict the data previously obtained in Wistar rats, where deficits were observed in almost all adult offspring affected by the prenatal immune challenge [35,36,37]. Interactions of multiple genetic (species and strain) and environmental factors [97] may cause variations in the PPI test. Also, the individual’s risk or resilience to neuroinflammatory disorders as a phenomenon dependent on early life experiences was suggested by Williamson et al. [98].

Researchers have proposed that MIA creates a long-term and latent vulnerability to the development of schizophrenia symptoms that are only unmasked by insults occurring later in life [99,100]. Therefore, we introduced a “second hit” in the form of the acute systemic LPS administration at PND120. The “two-hit” model of schizophrenia triggered PPI deficits in the MIA non-responsive offspring. Although the effects induced by the second challenge, as observed in the PPI test, are difficult to interpret, they may reflect sensitization formed in response to MIA. To date, only a few studies have examined the acute effect of LPS on the acoustic startle response and PPI, showing no impact of LPS administration on those parameters [101,102]. Other studies established that adult rats treated with LPS displayed an altered startle response in a dose-dependent manner, while PPI was largely unaffected [103]. The effect of the acute LPS treatment on PPI is ambiguous. Therefore, the impact of different intervals between administration and testing, doses, and species and strain differences on the reaction to LPS [104,105] and on the startle response [106,107,108] should be underscored.

An intriguing observation from our study is that MIA in adult offspring, in addition to behavioural modulation, impaired the neuron-microglia crosstalk, as evidenced by changes in the CD200–CD200R and CX3CL1–CX3CR1 axes. To the best of our knowledge, we are the first group to report this finding in the neurodevelopmental model of schizophrenia in Sprague-Dawley rats. The majority of MIA-evoked changes were related to the frontal cortex, in which a decrease in the CD200R level has been shown. The diminished level of CD200R was observed both in the responsive and non-responsive animals. However, in the non-responsive offspring, the upregulation of *Cd200* and *Cd200r* expression was simultaneously observed. Therefore, the impact of MIA on the disturbances in the post-translational processing of proteins or accelerated degradation of enzymatic proteins and the presence of compensatory mechanisms cannot be excluded. In line with this phenomenon, after the “second hit”, the changes in cortical CD200R level were only found in the non-responsive rats, for which we also detected the dysfunction of the CD200–CD200R axis in the hippocampus. Notably, disturbances in the CD200R expression do not result from changes in the number of microglial cells because we have shown that neither MIA nor the “second hit” affected the IBA1 levels. 

In agreement with the above-mentioned data, the disturbances in the CX3CL1–CX3CR1 network, although less pronounced, were mainly present in the non-responsive animals. More precisely, in the frontal cortex, MIA reduced the CX3CL1 level (like after an acute LPS treatment), while diminished the CX3CR1 level in the hippocampus. In the frontal cortex of the non-responsive offspring, we revealed a reduced level of CX3CR1 after the “second hit”, compared to the MIA model and the control offspring after the additional LPS treatment. On the other hand, in the responsive offspring, the suppressive effect of the “second hit” on the ligand expression was found in both brain areas. It seems that the changes we observed in the CD200–CD200R and CX3CL1–CX3CR1 pathways mainly in the non-responsive offspring may have a greater potential to shift microglial activity towards the pro-inflammatory phenotype and/or priming of microglia.

Systems controlling neuron–microglia communication exert a crucial impact on the various microglial functions [34,109,110,111]. The CX3CL1–CX3CR1 axis regulates neurodevelopmental processes, including neuronal survival [30] and synaptic pruning [112]. Disturbances in this pathway have also been shown to be involved in behavioural abnormalities [113]. *Cx3cr1*-/- knockout mice exhibit the hyperactive phenotype [114], a deficiency associated with impaired long-lasting connectivity and social behaviour [115], and changes in fear and anxiety [113]. Interestingly, unconditioned reflexive responses regulated by sensorimotor pathways are not impacted by the CX3CR1 deficiency in mice [113]. These results do not exclude the possibility that the MIA-evoked disturbances of the CX3CL1–CX3CR1 system in the non-responsive offspring, observed in our study, potentiate the susceptibility to induction of the behavioural deficits, consistent with the “two-hit” hypothesis of schizophrenia [116].

In the context of our research, the observation that both CD200–CD200R and CX3CL1–CX3CR1 dyads are neuroinflammatory “off” signals for microglia is of crucial significance [117,118]. The dysfunction of the CD200–CD200R signalling pathway exaggerates the pro-inflammatory response of microglia to immune challenge [119] and leads to a prolonged inflammatory response [120,121]. Microglia-mediated damage in schizophrenia-sensitive regions, such as the frontal cortex and hippocampus, may directly lead to cognitive and negative symptoms, as well as a number of the changes in brain structures associated with schizophrenia. The loss of cortical control may also explain the disinhibition of subcortical dopamine signalling, causing positive psychotic symptoms [122]. Thus, an understanding of the mechanisms regulating the CD200–CD200R axis, which controls microglial activity, is crucial. Data postulate that pro-inflammatory cytokines exert negative regulatory potential, while anti-inflammatory cytokines have a positive regulatory effect on this dyad. In our study, the CD200R deficit was accompanied by an increased level of IL-6 in the frontal cortex of the responsive offspring. Excessive IL-6 release inhibits dendrite development on cortical neurons in rats [123] and modulates the generation of neural progenitor cells in the adult subventricular zone, during neurogenesis [124]. In adult neurons, an increased IL-6 level has been associated with the dysfunction of GABAergic parvalbumin-containing inhibitory neurons [40,125] leading to schizophrenia-like behavioural disturbances. Hence, the role of IL-6 in inducing behavioural deficits is long-lasting and occurs over time [37,126]. In the Wistar rat MIA model, we showed elevated IL-6 level that preceded the appearance of behavioural deficits [37]. The study by Borrell et al. [58] showed raised serum levels of IL-1β, IL-6, and IL-2 in a time course of two hours after subcutaneous LPS administration to female rats, as well as IL-6 and IL-2 in adult MIA offspring. Additionally, an acute IL-6 treatment induced a deficit in sensorimotor gating in mice [62]. It seems that the MIA-induced changes in the pro-inflammatory profile (IL-1β, TNF-α and IL-6) potentially affect neurodevelopmental processes in the offspring in a uniform manner, while the increased level of IL-6 present in the adult responsive offspring was a factor sufficient to induce the PPI deficits. This cytokine, a negative regulator of the CD200–CD200R axis, mediates the behavioural response to the “second hit” associated with microglial “priming” and the induction of various inflammatory events, as well as elevated cortisol production. Even if the behavioural impairment is not initially apparent in our study in the non-responsive offspring, we were unable to exclude the possibility that a single LPS injection in adulthood triggered a series of substantial disturbances. Comparatively, Williamson et al. [98] showed that early-life infection can change microglial function within the brain and exaggerate the response of microglia to a subsequent challenge, e.g., “second hit” with LPS.

IL-6 is involved in the pronounced age-dependent priming of microglia [127]. The lack of an overt pro-inflammatory profile, despite a shift in the immuno-phenotype, is a hallmark of primed microglia. Upon exposure to a pro-inflammatory stimulus, primed microglia exhibit an excessive inflammatory response [23], suggesting that inhibitory control over microglia has been attenuated. The disruption of CD200–CD200R signalling potentiates the microglial pro-inflammatory response to subsequent immune challenges [119,128]. The reduced CX3CR1 level may drive the neuroinflammatory priming of microglia in aged mice [129]. In our study, in the non-responsive offspring, the additional LPS challenge excessively upregulated *Il-6* expression in the tested brain areas and the *Tnf-α* level in the hippocampus compared with the responsive and the control animals injected with the “second hit”. The IL-6 release is not specific to microglia exclusively [130], and thus it cannot be excluded that the increase in *Il-6* level was related in part to the secretion of this cytokine by astrocytes. We also showed the upregulation of pro-inflammatory microglial markers *Cd40* and *iNos* in the non-responsive offspring. Thus, MIA-induced dampening of the pathways through which neurons inhibit microglia might produce a microenvironment permissive to a prolonged “primed” pro-inflammatory response in the brain. 

IL-4, which is a positive regulator of the CD200–CD200R axis [131], exerts procognitive and protective effects on the CNS [22,132,133]. This cytokine, acting via the IL-4 receptor, triggers downstream processes that induce anti-inflammatory activity by inhibiting the NF-κB pathway, while STAT6 phosphorylation results in a shift to anti-inflammatory microglia polarization and upregulation of the *Arg1* expression [134]. A potential beneficial role for ARG1 in inflammation has been suggested to involve competition with iNOS, as both of these factors utilize L-arginine as a substrate. As reported by Yi et al. [134], the *Arg1* level was significantly decreased by CD200R knock-down. In our study, we observed a reduced expression of *Arg1* in the hippocampus of the responsive offspring after MIA. Although we did not find concomitant changes in the hippocampal levels of IL-4 and CD200R, this finding indicated that anti-inflammatory processes in the responsive animals were somehow impaired, allowing for the manifestation of behavioural disturbances. 

On the other hand, in the frontal cortex of the non-responsive offspring, we observed the MIA-evoked increase in the level of IL-4. The additional LPS treatment boosted the *Il-4* expression in comparison with the control and the responsive animals after the “second hit”, which may indicate an alternative mechanism of microglia priming in the non-responsive offspring. Therefore, in the non-responsive offspring, IL-4 may have suppressed the MIA-evoked changes, while their emergence as behavioural and microglia priming manifestations may have required the “second hit”.

## 5. Conclusions

Taken together, in Sprague-Dawley rats, the maternal immune challenge resulted in behavioural deficits in adult offspring. The application of the PPI test allowed us to distinguish two distinct groups among the animals, namely, responsive and non-responsive behavioural phenotypes. Our study is the first to raise the intriguing possibility that the MIA-induced disturbances in the CD200–CD200R system, as well as its negative (IL-6) and positive (IL-4) immune regulators, determine the manifestation of the schizophrenia-like behavioural disturbances. The reduced CD200R-mediated inhibitory drive appears to be involved in priming the microglia of the non-responsive offspring, as revealed by the “second hit” in the form of acute systemic LPS treatment in adulthood. Therefore, the role of neuron–microglia (CD200–CD200R) communication in pathogenesis and susceptibility to schizophrenia-like deficits should be underscored.

## Figures and Tables

**Table 1:** The effect of MIA on the social (aggressive and non-aggressive) behaviour of adult male Sprague-Dawley offspring, measured in the test of social interactions. *n* = 6 in each group. The results are presented as the means ± SEM.

Group	Type of Social Interaction			
	Aggressive		Non-Aggressive	
	Number of Events	Time [s]	Number of Events	Time [s]
control	4.83 ± 2.17	11.33 ± 4.52	29.67 ± 6.71	82.83 ± 15.92
MIA	2.00 ± 1.63	4.50 ± 3.18	42.33 ± 7.10	124.67 ± 20.49

**Table 2:** The effect of MIA on the prepulse inhibition of the acoustic startle response (PPI) in male Sprague-Dawley offspring at PND30 and PND60. *n* = 8–11 in each group. The results are presented as the means of the percentage of PPI (%PPI) induced by each prepulse intensity ± SEM. Data were calculated based on the average startle amplitudes (AVGs). * *p* < 0.05 vs. control group.

Prepulse Intensity	Group			
	PND30		PND60	
	Control	MIA	Control	MIA
70 dB	16.21 ± 9.67	25.08 ± 5.27	38.07 ± 3.95	44.66 ± 5.01
75 dB	21.45 ± 8.45	43.85 ± 3.22 *	58.60 ± 4.22	59.33 ± 4.02
80 dB	33.93 ± 9.45	37.34 ± 5.69	52.00 ± 4.25	61.12 ± 3.53

## Supplementary Materials

The following are available online at https://www.mdpi.com/2073-4409/9/7/1676/s1.
