# Supplementary material for: Maternal Immune Activation Sensitizes Male Offspring Rats to Lipopolysaccharide-Induced Microglial Deficits Involving the Dysfunction of CD200–CD200R and CX3CL1–CX3CR1 Systems
Source: Cells. 2020 Jul 12;9(7):1676. doi: 10.3390/cells9071676 (PMC7407118; doi:10.3390/cells9071676)
Supplement: Supplementary file 1 [file cells-09-01676-s001.pdf]

Supplementary materials

3. Results

3.1. *Exploratory activity*

- the total distance travelled: 2285.69 ± 84.80 control vs. 2573.32 ± 94.02 MIA, p = 0.0389 (Figure 2)
- the exploration in the second interval of the experiment: 456.81 ± 21.30 control vs. 562.34 ± 23.66 MIA, p = 0.0029 (Figure 2)

3.2. *Light-dark box test*

- the time spent in the light compartment: 54.18 ± 6.69 control vs. 34.87 ± 6.02 MIA, p = 0.0401 (Figure 3)
- the distance travelled in the light compartment: 1116.86 ± 147.79 control vs. 656.19 ± 116.68 MIA, p = 0.0162 (Figure 3)
- the time spent in the dark compartment: 545.82 ± 6.69 control vs. 565.14 ± 6.02 MIA, p = 0.0401 (Figure 3)
- the distance travelled in the dark compartment: 4538.70 ± 138.99 control vs. 4128.84 ± 118.53 MIA, p = 0.0299 (Figure 3)
- the average speed in the dark compartment: 8.42 ± 0.31 control vs. 7.44 ± 0.27 MIA, p = 0.0213 (Figure 3)

3.3. *Forced swim test*

No significant changes (Figure 4).

3.4. *Social interaction test*

No significant changes (Table 1).

3.5. *Prepulse inhibition of the acoustic startle response*

- PPI, the 75 dB prepulse, the animals at PND30: 21.45 ± 8.45 control vs. 43.85 ± 3.22 MIA, p = 0.0157 (Table 2)
- PPI, the 70 dB prepulse, the animals at PND100: 60.53 ± 2.60 control vs. 44.43 ± 2.93 MIA responsive, p = 0.0008; 60.53 ± 2.60 control vs. 71.32 ± 2.77 MIA non-responsive, p = 0.0126 (Figure 5)
- PPI, the 75 dB prepulse, the animals at PND100: 76.18 ± 2.38 control vs. 66.39 ± 3.39 MIA responsive, p = 0.0252; 76.18 ± 2.38 control vs. 83.17 ± 1.59 MIA non-responsive, p = 0.0475 (Figure 5)
- PPI, the 70 dB prepulse, the animals at PND120: 68.27 ± 5.02 MIA non-responsive + vehicle vs. 26.13 ± 11.04 MIA non-responsive + LPS, p = 0.0005 (Figure 5)

– PPI, the 75 dB prepulse, the animals at PND120: 61.77 ± 4.18 control + LPS vs. 77.24 ± 5.89 MIA responsive + LPS, p = 0.0182; 61.32 ± 5.24 MIA non-responsive + LPS vs. 77.24 ± 5.89 MIA responsive + LPS, p = 0.0335; 83.46 ± 1.61 MIA non-responsive + vehicle vs. 61.32 ± 5.24 MIA non-responsive + LPS, p = 0.0042; 73.49 ± 3.24 control + vehicle vs. 61.77 ± 4.18 control + LPS, p = 0.0440 (Figure 5)

3.6. The mRNA expression of the Cx3cl1, Cx3cr1, Cd200 and Cd200r in the frontal cortices and hippocampi of adult male offspring

– Frontal cortex, *Cd200*: 1.02 ± 0.08 control + vehicle vs. 1.31 ± 0.07 MIA non-responsive + vehicle, p = 0.0313; 0.99 ± 0.11 MIA responsive + vehicle vs. 1.31 ± 0.07 MIA non-responsive + vehicle, p = 0.0345 Figure 6)

– Frontal cortex, *Cd200r*: 1.08 ± 0.18 control + vehicle vs. 1.67 ± 0.16 MIA non-responsive + vehicle, p = 0.0329; 1.03 ± 0.25 MIA responsive + vehicle vs. 1.67 ± 0.16 MIA non-responsive + vehicle, p = 0.0340; 1.67 ± 0.16 MIA non-responsive + vehicle vs. 0.83 ± 0.12 MIA non-responsive + LPS, p = 0.0050) (Figure 6)

– Frontal cortex, *Cx3cl1*: 1.12 ± 0.09 control + LPS vs. 0.93 ± 0.10 MIA responsive + LPS, p = 0.0456 (Figure 6).

3.7. Levels of the CX3CL1, CX3CR1, CD200 and CD200R proteins in the frontal cortices and hippocampi of adult male offspring

– Frontal cortex, CX3CL1: 0.16 ± 0.02 control + vehicle vs. 0.11 ± 0.02 MIA non-responsive + vehicle, p = 0.0382; 0.16 ± 0.02 control + vehicle vs. 0.09 ± 0.01 control + LPS, p = 0.0011 (Figure 7)

– Frontal cortex, CX3CR1: 31.67 ± 3.12 MIA non-responsive + vehicle vs. 23.22 ± 2.35 MIA non-responsive + LPS, p = 0.0137; 29.09 ± 1.12 control + LPS vs. 23.22 ± 2.35 MIA non-responsive + LPS, p = 0.0440 (Figure 7)

– Frontal cortex, CD200R: 25.17 ± 0.64 control + vehicle vs. 21.61 ± 0.35 MIA responsive + vehicle, p = 0.0423; 25.17 ± 0.64 control + vehicle vs. 20.52 ± 0.55 MIA non-responsive + vehicle, p = 0.0059; 25.17 ± 0.64 control + vehicle vs. 20.42 ± 0.73 control + LPS, p = 0.0011 (Figure 7)

– Hippocampus, CX3CR1: 15.08 ± 0.90 control + vehicle vs. 12.68 ± 0.83 MIA non-responsive + vehicle, p = 0.0491 (Figure 7)

– Hippocampus, CD200: 73.17 ± 2.98 control + vehicle vs. 62.16 ± 2.00 MIA non-responsive + vehicle, p = 0.0111 (Figure 7)

– Hippocampus, CD200R: 19.69 ± 1.44 MIA responsive + vehicle vs. 15.66 ± 0.91 MIA non-responsive + vehicle, p = 0.0348 (Figure 7)

– Hippocampus, CX3CL1: 0.15 ± 0.03 MIA responsive + vehicle vs. 0.08 ± 0.02 MIA responsive + LPS, p = 0.0245 (Figure 7)

3.8. The IBA1 levels in the frontal cortices and hippocampi of adult male offspring

No significant changes (Figure 8).

3.9. The mRNA expression of the microglial markers in the frontal cortices and hippocampi of adult male offspring

- Frontal cortex, *Cd40*: 0.89 ± 0.14 MIA responsive + vehicle vs. 3.87 ± 0.60 MIA responsive + LPS, p = 0.0016; 1.16 ± 0.09 MIA non-responsive + vehicle vs. 4.71 ± 1.14 MIA non-responsive + LPS, p = 0.0011; (Figure 9)
- Frontal cortex, *iNos*: 2.28 ± 0.35 MIA responsive + vehicle vs. 21.56 ± 3.91 MIA responsive + LPS, p = 0.0057; 0.88 ± 0.34 MIA non-responsive + vehicle vs. 38.34 ± 2.32 MIA non-responsive + LPS, p < 0.0001; 21.56 ± 3.91 MIA responsive + LPS vs. 38.34 ± 2.32 MIA non-responsive + LPS, p = 0.0019; 25.51 ± 3.85 control + LPS vs. 38.34 ± 2.32 MIA non-responsive + LPS, p = 0.0067; 1.03 ± 0.19 control + vehicle vs. 25.51 ± 3.85 control + LPS, p < 0.0001 (Figure 9)
- Frontal cortex, *Il-1β*: 0.97 ± 0.10 MIA responsive + vehicle vs. 4.24 ± 1.47 MIA responsive + LPS, p = 0.0239; 1.19 ± 0.30 MIA non-responsive + vehicle vs. 6.59 ± 1.43 MIA non-responsive + LPS, p = 0.0012; 1.04 ± 0.12 control + vehicle vs. 5.13 ± 0.76 control + LPS, p = 0.0003 (Figure 9)
- Frontal cortex, *Tnf-α*: 1.00 ± 0.11 MIA responsive + vehicle vs. 5.00 ± 0.94 MIA responsive + LPS, p = 0.0212; 1.61 ± 0.20 MIA non-responsive + vehicle vs. 9.95 ± 2.18 MIA non-responsive + LPS, p < 0.0001; 5.00 ± 0.94 MIA responsive + LPS vs. 9.95 ± 2.18 MIA non-responsive + LPS, p = 0.0036; 6.52 ± 0.67 control + LPS vs. 9.95 ± 2.18 MIA non-responsive + LPS, p = 0.0129; 1.03 ± 0.09 control + vehicle vs. 6.52 ± 0.67 control + LPS, p = 0.0001 (Figure 9)
- Frontal cortex, *Il-6*: 0.81 ± 0.09 MIA responsive + vehicle vs. 2.25 ± 0.40 MIA responsive + LPS, p = 0.0172; 0.96 ± 0.17 MIA non-responsive + vehicle vs. 3.90 ± 0.91 MIA non-responsive + LPS, p = 0.0001; 2.25 ± 0.40 MIA responsive + LPS vs. 3.90 ± 0.91 MIA non-responsive + LPS, p = 0.0068; 2.13 ± 0.23 control + LPS vs. 3.90 ± 0.91 MIA non-responsive + LPS, p = 0.0020; 1.07 ± 0.16 control + vehicle vs. 2.13 ± 0.23 control + LPS, p = 0.0216 (Figure 9)
- Frontal cortex, *MhcII*: 4.90 ± 0.67 control + LPS vs. 2.72 ± 0.51 MIA non-responsive + LPS, p = 0.0324; 1.10 ± 0.37 control + vehicle vs. 4.90 ± 0.67 control + LPS, p = 0.0001 (Figure 9)
- Frontal cortex, *Cd40*: 1.03 ± 0.10 control + vehicle vs. 4.46 ± 0.53 control + LPS, p < 0.0001 (Figure 9)
- Frontal cortex, *Il-4*: 0.87 ± 0.13 control + LPS vs. 1.98 ± 0.38 MIA non-responsive + LPS, p = 0.0047; 0.91 ± 0.12 MIA responsive + LPS vs. 1.98 ± 0.38 MIA non-responsive + LPS, p = 0.0127 (Figure 10)
- Frontal cortex, *Tgf-β*: 1.01 ± 0.05 control + vehicle vs. 1.26 ± 0.05 control + LPS, p = 0.0157 (Figure 10)
- Hippocampus, *MhcII*: 0.72 ± 0.08 MIA responsive + vehicle vs. 1.47 ± 0.27 MIA responsive + LPS, p = 0.0229; 0.56 ± 0.11 MIA non-responsive + vehicle vs. 1.50 ± 0.18 MIA non-responsive + LPS, p = 0.0017 (Figure 9)
- Hippocampus, *Cd40*: 0.87 ± 0.16 MIA responsive + vehicle vs. 3.15 ± 0.44 MIA responsive + LPS, p = 0.0034; 0.90 ± 0.15 MIA non-responsive + vehicle vs. 4.14 ± 0.67 MIA non-responsive + LPS, p < 0.0001; 2.85 ± 0.52 control + LPS vs. 4.14 ± 0.67 MIA non-responsive + LPS, p = 0.0413; 1.08 ± 0.22 control + vehicle vs. 2.85 ± 0.52 control + LPS, p = 0.0045 (Figure 9)
- Hippocampus, *Tnf-α*: 0.93 ± 0.19 MIA responsive + vehicle vs. 4.62 ± 0.82 MIA responsive + LPS, p < 0.0001; 1.05 ± 0.16 MIA non-responsive + vehicle vs. 4.91 ± 0.62 MIA non-responsive + LPS, p < 0.0001; 1.04 ± 0.12 control + vehicle vs. 3.62 ± 0.50 control + LPS, p = 0.0003 (Figure 9)
- Hippocampus, *Il-6*: 0.69 ± 0.12 MIA responsive + vehicle vs. 1.89 ± 0.12 MIA responsive + LPS, p = 0.0001; 0.77 ± 0.04 MIA non-responsive + vehicle vs. 2.24 ± 0.34 MIA non-responsive + LPS, p < 0.0001; 1.42 ± 0.12 control + LPS vs. 2.24 ± 0.34 MIA non-responsive + LPS, p = 0.0017 (Figure 9)
- Hippocampus, *iNos*: 0.37 ± 0.10 MIA non-responsive + vehicle vs. 5.69 ± 1.76 MIA non-responsive + LPS, p = 0.0014; 0.99 ± 0.25 control + vehicle vs. 5.43 ± 1.10 control + LPS, p = 0.0008 (Figure 9)
- Hippocampus, *Il-1β*: 0.72 ± 0.05 MIA non-responsive + vehicle vs. 4.24 ± 1.02 MIA non-responsive + LPS, p = 0.0010; 1.04 ± 0.13 control + vehicle vs. 4.36 ± 0.89 control + LPS, p = 0.0005 (Figure 9)

- Hippocampus, *Arg1*: 1.02 ± 0.09 control + vehicle vs. 0.60 ± 0.07 MIA responsive + vehicle, $p = 0.0058$; 0.60 ± 0.07 MIA responsive + vehicle vs. 0.99 ± 0.08 MIA responsive + LPS, $p = 0.0132$; 0.60 ± 0.07 MIA responsive + vehicle vs. 0.74 ± 0.12 MIA non-responsive + vehicle, $p = 0.0452$
- Hippocampus, *Igf-1*: 1.01 ± 0.06 control + vehicle vs. 1.30 ± 0.23 MIA responsive + vehicle, $p = 0.0236$; 1.30 ± 0.23 MIA responsive + vehicle vs. 0.91 ± 0.07 MIA responsive + LPS, $p = 0.0052$
- Hippocampus, *Il-4*: 1.11 ± 0.19 MIA responsive + vehicle vs. 0.39 ± 0.09 MIA responsive + LPS, $p = 0.0123$; 0.39 ± 0.09 MIA responsive + LPS vs. 1.02 ± 0.10 MIA non-responsive + LPS, $p = 0.0205$ (Figure 10)

3.10. Levels of the IL-6 and IL-4 proteins in the frontal cortices and hippocampi of adult male offspring

- Frontal cortex, IL-6: 9.18 ± 0.49 control + vehicle vs. 12.59 ± 1.98 MIA responsive + vehicle, $p = 0.0457$
- Frontal cortex, IL-4: 3.68 ± 0.21 control + vehicle vs. 5.00 ± 0.50 MIA non-responsive + vehicle, $p = 0.0029$; 3.76 ± 0.12 control + LPS vs. 5.03 ± 0.50 MIA non-responsive + LPS, $p = 0.0042$ (Figure 11)
